# Genetic Diversity, Pathogenicity and Pseudorecombination of Cucurbit-Infecting Begomoviruses in Malaysia

**DOI:** 10.3390/plants10112396

**Published:** 2021-11-06

**Authors:** Yu-Jeng Chen, Hsuan-Chun Lai, Chung-Cheng Lin, Zhuan Yi Neoh, Wen-Shi Tsai

**Affiliations:** 1 Department of Plant Medicine, National Chiayi University, Chiayi City 600355, Taiwan; v8645017@gmail.com (Y.-J.C.); laichun71@gmail.com (H.-C.L.); neohzhuanyi@yahoo.com (Z.Y.N.); 2 Clover Seed Company Ltd., Hong Kong; cc-lin@cloverseed.cn

**Keywords:** *Squash leaf curl China virus*, *Tomato leaf curl New Delhi virus*, agroinoculation, polymerase chain reaction, host range

## Abstract

Cucurbits are important crops in the world. However, leaf curl disease constrains their production. Here, begomovirus diversity and pathogenicity associated with the disease in Malaysia were studied based on 49 begomovirus-detected out of 69 symptomatic plants from seven cucurbit crops in 15 locations during 2016 and 2017. The presence of *Squash leaf curl China virus* (SLCCNV) and *Tomato leaf curl New Delhi virus* (ToLCNDV) were confirmed by virus detection by polymerase chain reaction, viral DNA sequence analysis and specific detection of the viral components. ToLCNDV Malaysian isolates were further distinguished into strains A, B, C and D. Virus co-infection was detected in bitter gourd, bottle gourd and squash. Among them, eight bitter gourd samples were detected without SLCCNV DNA-A. However, one bottle gourd and five squash samples were without ToLCNDV DNA-B. Pseudorecombination of ToLCNDV DNA-A and SLCCNV DNA-B was detected in two bitter gourd samples. The pathogenic viruses and pseudorecombinants were confirmed by agroinoculation. The viral DNA-B influencing on symptomology and host range was also confirmed. The results strengthen the epidemic of cucurbit-infecting begomovirus in Malaysia as well as Southeast Asia. Especially, the natural pseudorecombinant of begomovirus that extends host range and causes severe symptom implies a threat to crops.

## 1. Introduction

Cucurbits are important economic crops, and their growing acreage has reached 7.89 million hectares (ha) with production of more than 238.6 million tons worldwide, with Asian countries being the major contributors [1]. However, leaf curl disease caused by whitefly-transmitted begomoviruses constrains cucurbit production [2,3,4]. Symptoms of cucurbit leaf curl disease include the leaf yellowing, mosaic and curling, and diseased plant may also reveal symptoms of vein swelling, yellow chlorotic spot and plant stunting [5,6,7]. Based on the demarcation criteria of nucleotide sequence identity 91% for the begomovirus species, more than 15 cucurbit-infecting begomovirus species were identified [8]. Three begomoviruses, *Tomato leaf curl New Delhi virus* (ToLCNDV), *Squash leaf curl China virus* (SLCCNV) and *Squash leaf curl Philippines virus* (SLCuPV) were frequently associated with cucurbit leaf curl disease in Asia [9,10,11]. All contain bipartite genome (DNA-A and -B). So far, ToLCNDV was identified on more than 15 cucurbit crops distributed in nine countries [10]. SLCCNV was identified on more than six cucurbit crops in eight countries [12,13,14,15,16,17,18]. The SLCuPV was first identified in the Philippines and most closely related to the SLCCNV (88% nucleotide identity of DNA-A) [5]. Consequentially, the virus was found in the Taiwan [19]. SLCuPV has a restricted host range within the *Cucurbitaceae* [20].

The genomic components of begomovirus could be evolved rapidly through mutation, recombination and pseudorecombination [21]. Pseudorecombination only occurs in viruses having segmented genomes [22]. A pseudorecombination can be defined as an exchange of homologous segments between two related virus isolates or species during the mixed infection in host cell. However, the potential pseudorecombination could be limited to specific portions of the viral genome [23]. The begomovirus recombination or pseudorecombination may occur among isolates, species and even across genera [24,25,26,27]. As a result, it will be a serious problem when the virus reveals new pathological properties, such as causing more severe symptom, increasing alternative hosts and affecting plant resistance [24,25,26,27]. So far, the begomovirus pseudorecombination occurred naturally in *Cotton leaf crumple virus* (CLCrV), a whitefly-transmitted bipartite begomovirus causing a cotton disease widely [28]. The CLCrV isolates, Arizona (AZ) or Sonora (SON), infected cotton plants and induced typical leaf crumple symptom and their DNA-A components shared 97.9% nucleotide identity; however, the common region (CR) of viral DNA-A and DNA-B components contains low nucleotide identity (77%). The CLCrV DNA-B was grouped in a distinct cluster suggesting that CLCrV genome may consider as a pseudorecombinant [28].

So far, the leaf curl disease of cucurbit crops is distributed worldwide including Southeast Asia [2,5,6,7]. However, the identification of cucurbit begomovirus is limited in Malaysia. Here, the genetic diversity and pathogenicity of cucurbit-infecting begomoviruses in Malaysia was studied, and the pseudorecombination assay of the pathogenic viruses was conducted as well.

## 2. Results

### 2.1. Virus Detection

In 2016, eight out of 11 cucurbit samples collected in East Malaysia were identified with the presence of *Begomovirus* (Table 1). In 2017, begomovirus was detected in 15 out of 16 samples collected from Kuching, Sarawak, Malaysia and in 26 out of 42 samples collected from Kulai, Johor, Malaysia (Table 1). In total, the begomovirus was detected in nine bitter gourd, one bottle gourd, 13 cucumber, one oriental melon, eight squash, 14 ridge gourd, and three wax gourd samples. Viral DNA-B was also detected in all begomovirus positive samples (Table 1). Viruses associated satellite DNAs were not detected in all begomovirus positive samples (data not shown).

### 2.2. Virus DNA Sequence Analysis

**Figure 1:**
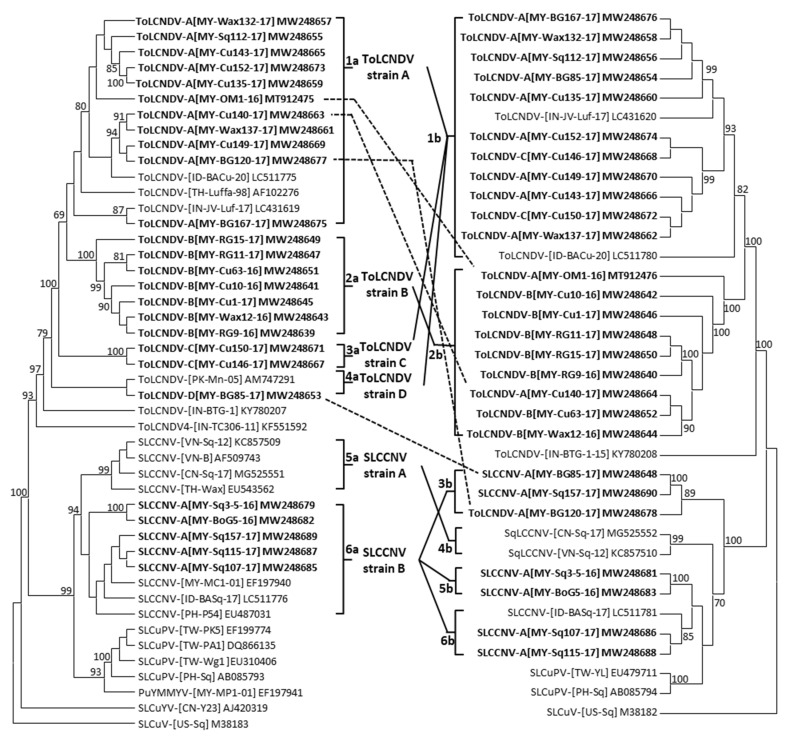
Phylogenetic tree obtained from the alignment of complete DNA-A and -B nucleotide sequences of cucurbit-infecting begomoviruses including viruses identified in this study (in bold) and selected sequences (listed in Table S2). Phylogenetic analysis was performed by Molecular Evolutionary Genetics Analysis software X (MEGA X) using the Muscle alignment and the neighbor-joining method options. The tree is rooted on the sequence of *Squash leaf curl virus* (SLCuV). The numbers at branch indicate the percentage of 1000 bootstrap replications.

In total, 26 full-length viral DNA-A and 27 full-length DNA-B sequences were determined (Figure 1, Table S3). All sequences contain the begomovirus conserved nonanucleotide sequence- TAATATT/AC in the stem-loop structure located in the intergenic region (IR). All DNA-A sequences were determined to contain two open reading frames (ORFs) (AV1 and AV2) in virion sense and four (AC1 to AC4) in complementary sense (Table S3). One each ORF in virion (BV1) and complementary sense (BC1) were also determined in all DNA-B sequences (Table S3). 

Based on the sequences analysis, DNA-A sequences including those newly identified in this study and retrieved from GenBank were grouped into six clusters (Figure 1).

Following demarcation criteria of nucleotide sequence identity 91% for the begomovirus species, the identified isolates in Clusters 1a, 2a, 3a and 4a were considered as ToLCNDV isolates (Table 2). Their full-length DNA-A sequences have >83.5% sequence identity with ToLCNDV isolates. All new identified isolates in Cluster 6a were considered as SLCCNV isolates, they revealed >89.0% sequence identity with other SLCCNV isolates. The virus isolates in each Cluster formed a distinct strain of the virus based on the demarcation criteria 94% sequence identity for begomovirus strain. The virus isolates in Cluster 1a composed of a distinct ToLCNDV strain A, they revealed 92.5–98.9% sequence identity to each other and 78.3–95.8% with other ToLCNDV isolates. The virus isolates in Cluster 2a composed of ToLCNDV strain B showing 93.9–99.5% sequence identity to each other and 78.3–92.9% with other ToLCNDV isolates. Both isolates in Cluster 3a composed of ToLCNDV strain C sharing 99.0% sequence identity and revealed 78.9–90.1% with other ToLCNDV isolates. Both isolates in Cluster 4a built up ToLCNDV strain D based on 94.5% sequence identity to each other and 80.8–88.0% with other ToLCNDV isolates. All virus isolates in Cluster 5a have 92.9–96.1% sequence identity to each other and grouped as SLCCNV strain A. The virus isolates in Cluster 6a composed of SLCCNV strain B showing 91.6–100.0% sequence identity to each other and 89.6–94.4% with SLCCNV-A isolates. The DNA-B sequences including newly identified full-length viral DNA-Bs and those retrieved from GenBank could be grouped into 6 Clusters (Figure 1). Viral DNA-B sequences in Cluster 1b and 2b were ToLCNDV DNA-Bs and revealed sequence identity 84.9–91.4% of DNA-Bs between two Clusters (Table 2). However, they shared >92.0% sequence identity within the Cluster 1b or 2b. Those in Cluster 3b, 4b, 5b and 6b were SLCCNV DNA-Bs sharing 70.9–85.1% sequence identity among the Clusters (Table 2). Those SLCCNV DNA-Bs within a Cluster shared >83.9% sequence identity to each other. 

Most of the viral DNA-As were identified with relative DNA-Bs (Figure 1). However, both ToLCNDV strain C isolates (Cu146 and Cu150) revealed viral components of DNA-As in Cluster 3a and of DNA-Bs in Cluster 1b. The viral DNA-As of two isolates (OM1 and Cu140) were grouped in ToLCNDV strain A and their component DNA-Bs were in Cluster 2b. Interestingly, based on the viral DNA-A analysis, the isolate BG120 was in ToLCNDV strain A and the isolate BG85 was in strain D. However, the viral DNA-Bs of both isolates were in Cluster 3b, the SLCCNV DNA-Bs (Figure 1).

### 2.3. Specific Detection of Cucurbit Begomovirus in Malaysia

All the samples detected successfully for the presence of specific virus component by PCR with the primer pairs: SLCCNV-SPAF/PAR1c715H for SLCCNV DNA-A, ToLCNDV-SPAF/PAR1c715H for ToLCNDV DNA-A, SLCCNV-SPBF/DNA-BC for SLCCNV DNA-B, and ToLCNDV-SPBF/DNA-BC for ToLCNDV DNA-B (Figure S1 and Table 2). The ToLCNDV DNA-A was detected in all begomovirus positive samples (Table 1). The ToLCNDV DNA-B was also presence in all, except two bitter gourd, one bottle gourd and five squash samples. However, the SLCCNV DNA-A was limited in one bottle gourd and seven squash samples. In addition, the SLCCNV DNA-B was present in eight bitter gourd, one bottle gourd and seven squash samples. Thirty-three, composing of one each bitter gourd and squash, and 13 cucumber, one oriental melon, 14 ridge gourd and three wax gourd samples showed only the presence of ToLCNDV DNAs without SLCCNV DNAs. Other 16 begomovirus positive samples were mixed with DNA components of ToLCNDV and SLCCNV. Interestingly, no SLCCNV DNA-A was detected in bitter gourd samples, whereas eight of them showed the presence SLCCNV DNA-B. Surprisingly, two bitter gourd samples from Kulai, Johor revealed the presence of only ToLCNDV DNA-A and SLCCNV DNA-B (Table 1). 

### 2.4. Pathogenicity of Cucurbit-Infecting Begomovirus Malaysian Isolates

**Figure 2:**
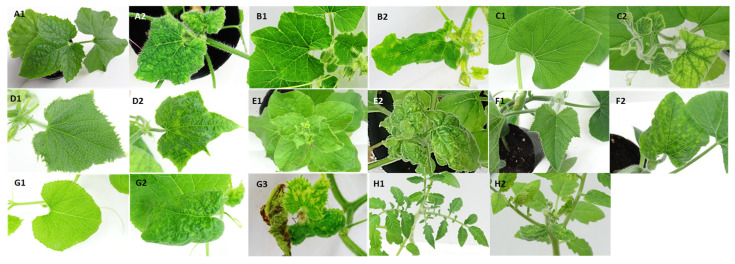
The plant symptoms induced by agroinoculation of infectious cucurbit-infecting begomoviral DNAs from Malaysia. Symptoms of leaf curling and blistering on wax gourd (*Lagenaria siceraria*) (**A2**), curling and yellow spots on pumpkin (*Cucurbita maxima*) (**B2**) and leaf curling and yellowing on bottle gourd (*Benincasa hispida*) (**C2**) were induced by agroinoculation with *Agrobacterium tumefaciens* LBA4404 containing the infectious DNA-A and B of SLCCNV-A[MY-Sq3-5-16], SLCCNV-A[MY-Sq115-17], SLCCNV-A[MY-Sq157-17], ToLCNDV-A[MY-OM1-16], ToLCNDV-B[MY-Wax12-16], natural pseudorecombinant ToLCNDV-D[MY-BG120-17] and artificial pseudorecombinant of ToLCNDV-B[MY-OM1-16] DNA-A with SLCCNV-A[MY-Sq3-5-16] DNA-B. Symptoms of leaf yellow spots on cucumber (*Cucumis sativus*) (**D2**), and leaf curling and blistering on tobacco (*Nicotiana benthamiana*) (**E2**) were induced by agroinoculation with all infectious ToLCNDV isolates, natural and the artificial pseudorecombinants. Symptoms of leaf curling yellow spots and blistering on squash (*Cucurbita moschata*) (**F2**) were induced by agroinoculation with all infectious SLCCNV isolates. Symptoms of leaf curling and blistering on oriental melon (*Cucumis melo*) (**G2**) were induced by agroinoculation with all infectious SLCCNV and ToLCNDV isolates, and artificial pseudorecombinant ToLCNDV-A[MY-BG120-17] DNA-A with ToLCNDV-A[MY-OM1-16] DNA-B. The additional symptoms of leaf yellow spots and necrosis (**G3**) was observed on oriental melon agroinoculated with infectious natural pseudorecombinant ToLCNDV-A[MY-BG120-17] and artificial pseudorecombinant ToLCNDV-A[MY-OM1-16] DNA-A with ToLCNDV-A[MY-BG120-17] DNA-B. Symptoms of leaf curling, yellow spots and blistering on tomato (*Solanum lycopersicum*) (**H2**) were induced by agroinoculation of infectious DNAs of natural pseudorecombinant ToLCNDV-D[MY-BG120-17] and artificial pseudorecombinant of ToLCNDV-B[MY-OM1-16] DNA-A with SLCCNV-A[MY-Sq3-5-16] DNA-B. (**A1**–**H1**) are symptomless plants of wax gourd, pumpkin, bottle gourd, cucumber, tobacco, squash, oriental melon and tomato, respectively, which were agroinoculated with pCAMBIA0380.

Totally, three infectious SLCCNV DNA-As and their associated infectious DNA-Bs were constructed (Table 3). Infectious ToLCNDV DNAs were also constructed of one isolate each of strain A, B and D. The infectious clones of natural pseudorecombinant ToLCNDV-A [MY-BG120-17] DNA-As were also constructed. The infectious DNA-A and DNA-B was mixed in a ratio of 1:1 of each combination and agroinoculated into the plants (Table 3). All infectious isolates of SLCCNV, ToLCNDV and the natural pseudorecombinant successfully infected bottle gourd, oriental melon, pumpkin and wax gourd with typical symptoms (Table 3, Figure 2). However, the infection rate is low (≤ 40%) on pumpkin via agroinoculation by SLCCNV-A[MY-Sq115-17], SLCCNV-A[MY-Sq157–17] and ToLCNDV-D[MY-BG85-17] (Table 3). Squash was infected by all SLCCNV isolates and revealed typical symptoms, but not by any of ToLCNDV isolate and the natural pseudorecombinant (Figure 2 and Table 3). The cucumber and tobacco can be infected by all ToLCNDV isolates and the natural pseudorecombinant, and revealed typical symptoms (Figure 2). However, the cucumber and tobacco plants were not successfully infected by SLCCNV isolates, while the virus can be detected only after agroinoculation (Table 3). The artificial pseudorecombinant of ToLCNDV-B[MY-Wax12-16] DNA-A with SLCCNV-A [MY-Sq3-5-16] DNA-B was able to infect plants of bottle gourd, cucumber, pumpkin, wax gourd and tobacco with typical symptoms (Figure 2). The virus of artificial pseudorecombinant was also detected in three out of thirteen squash plants, which were symptomless after agroinoculation. Interestingly, the tomato plant was only infected successfully by both natural and artificial pseudorecombinants (Table 3). Even the tomato was also infected by the ToLCNDV-A[MY-OM1-16] and ToLCNDV-B[MY-Wax12-16], but they revealed symptomless and low ratio of virus detection (Table 3). All virus infection was completed through virus detection by PCR in those agroinoculated plants (Table 3).

Compared with ToLCNDV-A[MY-OM1-16], the natural pseudorecombinant ToLCNDV-A[MY-BG120-17] can induce additional symptoms of yellow spots and necrosis on oriental melon (Figure 2). The symptoms on melon plants appeared at 7–10 days after agroinoculated with natural pseudorecombinant ToLCNDV-A[MY-BG120-17]. However, the symptoms emerged at 10–14 days after agroinoculated with ToLCNDV-A[MY-OM1-16]. Based on the high nucleotide sequence identity (>94.9%), the TATA motifs and the Rep binding site (GGCGTCTGGCGT) were determined in CR of both viral components of the ToLCNDV-A[MY-OM1-16] and the natural pseudorecombinant ToLCNDV-A[MY-BG120-17], the artificial pseudorecombination was also conducted by exchanging the infectious DNAs between both virus isolates. When oriental melon was co-inoculated with ToLCNDV-A[MY-OM1-16] DNA-A and ToLCNDV-A[MY-BG120-17] DNA-B, symptoms on all eight plants appeared also at 7–10 days after agroinoculation and revealed the additional symptoms of necrosis and yellow spots which is similar to those appeared on ToLCNDV-A[MY-BG120-17] inoculated plants. However, when the oriental melon was co-inoculated with ToLCNDV-A[MY-BG120-17] DNA-A and ToLCNDV-A[MY-OM1-16] DNA-B, the symptom type and the timing on four out of five inoculated plants were almost the same as those on ToLCNDV-A[MY-OM1-16]-inoculated plants. All virus infections were confirmed on those agroinoculated plants through virus detection by PCR (data not shown).

## 3. Discussion

Cucurbit leaf curl disease caused by whitefly-transmitted begomoviruses is one of the important cucurbit diseases. SLCCNV, SLCuPV and ToLCNDV are the major disease viruses in Southeast Asia. ToLCNDV have been recorded in India [29], Southeast Asia [3], Spain [30], the Mediterranean [31] and Taiwan [32]. SLCCNV is currently distributed in China [33], India [14,15,16,17] and Southeast Asia [12,13,34]. The SLCuPV was identified in the Philippines and Taiwan [5,19]. Here, two begomoviruses, ToLCNDV and SLCCNV, and their natural pseudorecombinants were identified in the Malaysian cucurbits based on the limited samples collected in 2016 and 2017. In total, 49 out of 69 symptomatic plants were detected with the presence of begomovirus. It may due to other cucurbit-infecting viruses, such as cucumber mosaic virus, potyviruses and tobamoviruses that can induce symptoms of mosaic, mottle, yellowing and plant stunting which are similar to the symptoms induced by begomoviruses on cucurbits [35]. Especially, cucurbit-infecting potyvirus has been found in Malaysia [36]. So far, ToLCNDV associated satellites were only observed in the virus causing tomato leaf curl disease [37], and no SLCCNV-associated satellite has been reported. This may explain why the virus-associated satellites were not detected in this study.

Furthermore, the virus DNA-As of ToLCNDV isolates are diverse and grouped into four strains (A, B, C and D), whereas SLCCNV Malaysian isolates are in strain B. However, viral DNA-Bs of SLCCNV isolates were more diverse than ToLCNDV isolates. ToLCNDV strain A contains virus isolates from India, Indonesia and Thailand, implicating the strain A isolates distributed widely in South and Southeast Asia, even in the East Malaysia area. This may be due to the fact that the virus isolates of ToLCNDV strain A have been identified from cucurbits in Thailand since the 1990s [10], adapted to cucurbit hosts and distributed in the regions. The ToLCNDV Malaysian isolate of strain D was from bitter gourd and grouped with a bitter gourd isolate previous detected in Pakistan (ToLCDNV-[PK-Mn-05], AM747291). However, the ToLCNDV was detected more frequently in Malaysia as compared to SLCCNV in this study (Figure 1, Table 1). 

ToLCNDV has broader cucurbit hosts than SLCCNV [37]. In this study, ToLCNDV was detected in more cucurbits than SLCCNV. In the limited numbers of Malaysian samples, the nature host of ToLCNDV includes bitter gourd, bottle gourd, cucumber, oriental melon, squash, ridge gourd and wax gourd, whereas SLCCNV was limited to bottle gourd and squash. However, by the specific detection of the virus components, those SLCCNV-detected bottle gourd and squash samples were also co-infected with ToLCNDV. Eight bitter gourd samples were detected with SLCCNV DNA-B, but not with SLCCNV DNA-A. However, ToLCNDV was found in those samples. Interestingly, two bitter gourd and six squash samples contain ToLCNDV DNA-A without ToLCNDV DNA-B, but associated with SLCCNV DNA-B. These are strong implication of the natural pseudorecombination between begomovirus species. Especially, the two bitter gourd samples that revealed the detection of ToLCNDV DNA-A and SLCCNV DNA-B (Table 1). The pseudorecombination may also occur between ToLCNDV strains such as the two isolates of strain A (OM1 and Cu140) are composed of DNA-As of strain A and DNA-Bs of strain B. So far, most begomovirus pseudorecombinants were confirmed by the experimental manner [24,25,26,27]. The natural pseudorecombinant has been possibly detected in ToLCNDV DNA-A with *Tomato leaf curl Palampur virus* (ToLCPalV) DNA-B in Pakistan. However, it was only confirmed by artificial pseudorecombination [27]. The natural pseudorecombinations occurred in cross species (ToLCNDV DNA-A with SLCCNV DNA-B) and within species (ToLCNDV DNA-A of strain A and DNA-B of strain B) were further confirmed by successful agroinoculation in this study. 

The pathogenicity of selected Malaysian isolates of SLCCNV and ToLCNDV were confirmed through agroinocluation and then the causal virus was detected by PCR. All Malaysian isolates of ToLCNDV and SLCCNV can successfully infect bottle gourd, oriental melon, pumpkin and wax gourd. The squash plants were efficiently infected by SLCCNV, not by ToLCNDV. However, cucumber and tobacco plants were effectively infected by ToLCNDV, not by SLCCNV. The SLCCNV caused symptoms of leaf curling and blistering on wax gourd, symptoms of leaf curling and blistering on oriental melon, leaf curling, blistering and yellow spots on pumpkin and squash. Those symptoms are similar to what was previously described [13,14,16,18]. The SLCCNV-infected bottle gourd exhibited symptoms of leaf curling and yellowing, which are similar to those described previously as of chlorotic mottling, mild curling and serious stunting [15]. The ToLCDNV can cause symptoms of leaf yellow spots on cucumber, and symptoms of leaf curling and yellow spots on pumpkin, leaf curling and yellowing on bottle gourd, and leaf curling and blistering on wax gourd. Those symptoms are similar to what was previously described [30,38,39,40,41]. ToLCNDV-infected oriental melon and tobacco plants developed symptoms of leaf curling and blistering, which are also similar to those previously described [42,43]; however, both plants infected by pseudorecombinant ToLCNDV-A[MY-BG120-17] exhibited severe symptoms of leaf curling, blistering, necrosis and yellow spots. Interestingly, only the natural pseudorecombinant ToLCNDV-A[MY-BG120-17] can successfully infect tomato plants and induced symptoms of leaf curling, blistering and yellow spots whereas neither other ToLCNDV nor SLCCNV natural isolates can. The symptom was similar to those described previously [7]. Nevertheless, the low rate of virus latent infection was confirmed in tomato plants which were agroinoculated with the ToLCNDV-A[MY-OM1-16] and ToLCNDV-A[MY-Wax12-16]. SLCCNV isolates can potentially infect cucumber and tobacco in a latent manner (Table 3). These hosts of virus latent infection could serve as a virus reservoir in the field and threaten the susceptible crops such as bottle gourd, oriental melon, pumpkin and squash.

Pseudorecombination of begomovirus could reportedly alter the host range [24,25]. This phenomenon was demonstrated by the natural pseudorecombinant ToLCNDV-A[MY-BG120-17] in this study. Only this natural pseudorecombinant can infect tomato successfully. The DNA-B component of natural pseudorecombinant ToLCNDV-A[MY-BG120-17] belongs to the group of SLCCNV DNA-B. The artificial pseudorecombinant of ToLCNDV-A[MY-Wax12-16] DNA-A with SLCCNV-A[MY-Sq3-5-16] DNA-B was confirmed to be able to infect tomato successfully. Yet, both original isolates ToLCNDV-A[MY-Wax12-16] and SLCCNV-A[MY-Sq3-5-16] cannot infect tomato well. This result implies that the viral DNA-B is the determinant responsible for the infection of tomato plants. The pseudorecombination of begomovirus is able to alter symptoms on host plants [26,27]. In this study, natural pseudorecombinant ToLCNDV-A[MY-BG120-17], an isolate of strain A, can infect oriental melon plants causing earlier and more severe symptoms with additional necrosis and yellow spots on leaves as compared to those infected by ToLCNDV-A[MY-OM1-16]. However, when the DNA-B was changed with ToLCNDV-A[MY-OM1-16] DNA-B, the symptoms developed were similar to ToLCNDV-A[MY-OM1-16]-infected oriental melon. Interestingly, when the oriental melon infected by ToLCNDV-A[MY-OM1-16] DNA-A with ToLCNDV-A[MY-BG120-17] DNA-B, the symptom developed was similar to those infected by the ToLCNDV-A[MY-BG120-17]. The results actually provided an evidence that the DNA-B of the natural pseudorecombinant ToLCNDV-A[MY-BG120-17] is associaated with the symptomatology. The virus DNA-B of *Pepper golden mosaic virus* (PepGMV) also played the key role on symptomatology confirmed by artificial pseudorecombination [25]. Further study is necessary to clarify the mechanism of ToLCNDV-A[MY-BG120-17] DNA-B involved in the host determination and in the symptom development. 

Based on the survey and limited cucurbit samples collected from Malaysia, SLCCNV, ToLCNDV and their pseudorecombinants were identified as the pathogens causing the leaf curl disease on major cucurbit crops in the country. Both SLCCNV and ToLCNDV were found in Eastern and Western Malaysia, even though SLCCNV was not detected in the samples collected from the Kuching area. ToLCNDV was found in all cucurbit crops and was classified into four strains due to its diversity. However, the SLCCNV was found majorly in bottle gourd and squash, and is more diversely associated with DNA-Bs. Based on the frequent detection of virus mixed-infection, this may compel the pseudorecombination of both begomoviruses [44]. In this study, the pseudorecombinant of ToLCNDV DNA-A with SLCCNV DNA-B was detected in field samples and confirmed by the pathogenicity study, which strongly exemplify the natural pseudorecombination of begomovirus. Due to the fact that the results were based on limited number of samples from Malaysia, therefore a comprehensive survey should be conducted in the future. The confirmed occurrence of both natural and artificial pseudorecombinants in the field increases the likelihood of host range expansion and more severe symptom incidence. Results presented in this study implicate that the increasing diversity of begomovirus is a threat to crop production in the Malaysia as well as in the Southeast Asia.

## 4. Materials and Methods

### 4.1. Sample Collection and Viral DNA Extraction

The diseased samples were collected from cucurbit plants exhibiting symptoms of mosaic, leaf curl and enation in 2016 and 2017 in Malaysia (Table 1). They were composed of 11 samples collected on six cucurbit species in two locations in East Malaysia in 2016, and 58 samples collected on six cucurbit species in 14 locations in Malaysia in 2017. One leaf sample was collected from each symptomatic cucurbit plant. All samples were dried by silica gel in Malaysia and imported into Taiwan following quarantine control. Viral DNA was extracted from approximately 50 mg of dried samples as previously described [45].

### 4.2. Begomoviral DNA Detection, Cloning and Sequence Analysis

The presence of begomovirus was confirmed by PCR using begomovirus DNA-A primers PAL1v1978RYNN and PAR1c715H [45]. The primer PAL1v1978RYNN was modified from PAL1v1978B (Table S1) [45]. The viral DNA-Bs of ToLCNDV and SLCCNV were detected by primer pair-DNA-BV/DNA-BC with annealing temperature of 52 °C. The virus associated satellite DNA was also detected as previously described [46]. Based on the location of samples collected, cucurbit hosts and homology analysis of partial viral DNA sequences, the full-length viral DNAs were amplified from selected samples (Figure 1). The full-length viral DNAs were amplified by PCR using designed abutting primer pair (Table S1) and the Q5^®^ High-Fidelity DNA Polymerase (New England BioLabs, USA). The PCR amplification was conducted as described previously [45]. The amplified viral DNAs were cloned by pGEM^®^-T Easy System (Promega, USA) and sequenced by auto-sequencing (Genomics, Taiwan). Obtained sequences were blasted with the available sequences in the GenBank using the program BLASTn. The nucleotide sequence identity of virus full-length sequences was generated by Cluster V method (DNASTAR, USA). Phylogenetic analysis was performed by Molecular Evolutionary Genetics Analysis software X (MEGA X) using the Muscle alignment and the neighbor-joining method options with 1,000 bootstrap replications [47]. The sequences of begomoviral DNAs were also retrieved from GenBank, NCBI for sequence analysis (Table S2). 

### 4.3. Virus Specific Detection

Based on the analysis of begomovirus sequences from Malaysia samples and those retrieved from GenBank, NCBI (Table S2), the primers for specific detection of SLCCNV and ToLCNDV were designed (Table S1). The primer pair-SLCCNV-SPAF/PAR1c715H was used for specific detection of SLCCNV DNA-As, whereas primer pair- ToLCNDV-SPAF/PAR1c715H was specific for ToLCNDV DNA-A. The amplicons of both primer pairs were about 1.5 kb in size (Figure S1). For specific detection of viral DNA-Bs, primer pairs-SLCCNV-SPBF /DNA-BC and ToLCNDV-SPBF/DNA-BC were used for specific detection of SLCCNV DNA-B and ToLCNDV DNA-B, respectively. The amplicons of both primer pairs are about 1.2 kb in size (Figure S1). 

### 4.4. Agroinfection of Infectious ToLCNDV and SLCCNV DNAs

Following the *Bam* HI and *Bgl* II digested DNA-A fragments (0.36 mer) of SLCCNV-A[MY-Sq3-5-16] was inserted into the binary vector pCAMBIA0380 (AF234290) [48], the infectious DNA-As were generated by the head–tail ligation of the full-length DNA-A which released from the recombinant plasmid by *Bgl* II digestion. Based on the *Eco* RI and *Pst* I digested DNA-B fragment (0.79 mer) was inserted into pCAMBIA0380, the infectious DNA-B of SLCCNV-A[MY-Sq3-5-16] was generated by the head–tail ligation of full-length DNA-B released by *Pst* I digestion. The *Bam* HI and *Bgl* II digested DNA-As fragments (0.59 mer) of SLCCNV-A[MY-Sq115-17] and SLCCNV-A[MY-Sq157-17] were inserted into the binary vector pCAMBIA0380, respectively. Consequently, the infectious DNA-A was generated by the head–tail ligation of the full-length DNA-A released by *Bam* HI. The *Eco* RI and *Nco* I fragments both of Sq115 (0.51 mer) and Sq157 (0.58 mer) DNA-Bs were also inserted into pCAMBIA0380, respectively. Consequently, the infectious DNA-B vector was generated following the head–tail ligation of full-length DNA-B released by *Nco* I. For infectious DNA-As of ToLCNDV-A[MY-OM1-16] and ToLCNDV-B[MY-Wax12-16], the *Eco* RI and *Pst* I digested fragments (0.28 mer) were inserted into the binary vector pCAMBIA0380, respectively, and then infectious DNA-As were generated by the head–tail ligation of the full-length DNA released by *Pst* I digestion. The infectious ToLCNDV-A[MY-BG120-17] DNA-A was constructed by insertion of *Bam* HI and *Pst* I digested fragments (0.27 mer) into pCAMBIA0380 following the head–tail ligation of the *Pst* I-released full-length DNA. The infectious ToLCNDV-D[MY-BG85-17] DNA-A was also constructed by insertion of *Bam* HI and *Sal* I digested fragments (0.31 mer) into pCAMBIA0380 following the head–tail ligation of the *Sal* I-released full-length DNA. For the infectious DNA-Bs of ToLCNDV-A[MY-OM1-16] and SLCCNV-A[MY-BG120-17], the *Eco* RI and *Nco* I fragments (0.51 mer of OM1 and 0.59 mer of BG120) were inserted into pCAMBIA0380, respectively. Consequently, infectious DNA-Bs were generated by the head–tail ligation of full-length DNA-B released by *Nco* I. The infectious ToLCNDV-B[MY-Wax12-16] DNA-B was generated by head–tail ligated dimer of full-length DNA-B released by *Nco* I. The infectious ToLCNDV-D[MY-BG85-17] DNA-B was generated by insertion of *Pst* I and *Nco* I digested fragment (0.44 mer) into pCAMBIA0380 following the head–tail ligation of the *Nco* I-released full-length DNA-B. All infectious DNAs were transferred respectively into *Agrobacterium tumefaciens* LBA4404 as previous description [45], and then cultured in YEP broth containing streptomycin (50 μg/ml) and kanamycin (50 μg/ml). Following incubation for 48 hours at 28 °C with 200 rpm shaking, the concentration of the bacterial culture was adjusted to OD_600nm_ = 1.0. Cucurbit plants including bottle gourd HV-026 (*Lagenaria siceraria cv.* Ever Happiness), squash HV-158 (*Cucurbita moschata*), cucumber HV-026 (*Cucumis sativus cv.* Merry Swallow), oriental melon HV-196 (*Cucumis melo cv.* Honey world), pumpkin HV-369 (*Cucurbita maxima*) and wax gourd HV-058 (*Benincasa hispida cv.* Benefit) (Know-You Seed CO., LTD., Taiwan) at three to five leaf stage were agroinoculated with infectious DNA combinations [45]. Tobacco (*Nicotiana benthamiana*) plants at four to six leaf stage and tomato (*Solanum lycopersicum cv.* ANT22) plants at five to eight leaf stage were also agroinoculated. Inoculated plants were grown in an insect-free greenhouse at 25 °C. Following the observation of symptom development for four weeks, the presence of viral DNA-A was confirmed by PCR using the primer pair PAL1v1978RYNN/PAR1c715H (Table S1).

## Figures and Tables

**Table 1:** Begomovirus DNA detection of cucurbit plant samples collected in Malaysia during 2016–2017.

Province	Location	Field	Year Collected	Crop	No. of Sample Collected	No. of Begomovirus Detected ^1^	Virus Specific Detection ^2^				Sample Number
							ToLCNDV		SLCCNV		
							DNA-A	DNA-B	DNA-A	DNA-B	
Sarawak	Sematan	1	2016	Oriental melon	1	1	1	1	0	1	OM1
		2		Squash	2	1	1	0	1	1	Sq3-5
		3		Bottle gourd	1	1	1	0	1	1	BoG5
	Kuching	4	2016	Ridge gourd	4	2	2	2	0	0	RG8 and RG9
		5		Cucumber	2	2	2	2	0	0	Cu10 and Cu11
		6		Wax gourd	1	1	1	1	0	0	Wax12
		7	2017	Cucumber	3	2	2	2	0	0	Cu1 and Cu3
		8		Ridge gourd	5	5	5	5	0	0	RG11 to 15
		9		Ridge gourd	7	7	7	7	0	0	RG26 to 32
		10		Cucumber	1	1	1	1	0	0	Cu63
Johor	Kulai	1	2017	Bitter gourd	1	1	1	1	0	1	BG85
		2		Bitter gourd	3	3	3	3	0	3	BG100 to 102
		3		Bitter gourd	5	3	2	0	0	2	BG120 and BG122
							1	1	0	0	BG121
				Squash	12	3	2	0	2	2	Sq107 and Sq115
							1	1	1	1	Sq112
		4		Wax gourd	1	1	1	1	0	0	Wax132
		5		Cucumber	1	1	1	1	0	0	Cu135
				Wax gourd	2	1	1	1	0	0	Wax137
		6		Cucumber	7	4	4	4	0	0	Cu140, Cu143, Cu144 and Cu146
		7		Cucumber	4	3	3	3	0	0	Cu149, Cu150 and Cu152
		8		Squash	4	4	2	0	2	2	Sq157 and Sq161
							1	1	1	1	Sq158
							1	1	0	0	Sq159
		9		Bitter gourd	2	2	2	2	0	2	BG166 and BG167
**Total**					**69**	**49**	**49**	**41**	**8**	**17**	

^1^ Number of samples were detected begomovirus positive by PCR using DNA-A primer pair-PAL1v1978RYNN/PAR1c715H and DNA-B primer pair-DNA-BV/DNA-BC; ^2^ The specific detection of virus DNAs were conducted by PCR using *Tomato leaf curl New Delhi virus* (ToLCNDV) DNA-A specific primer pair-ToLCNDV-SPAF/PAR1c715H, the ToLCNDV DNA-B specific primer pair-ToLCNDV-SPBF/DNA-BC, the *Squash leaf curl China virus* (SLCCNV) DNA-A specific primer pair-SLCCNV-SPAF/PAR1c715H, and the SLCCNV DNA-B specific primer pair-SLCCNV-SPBF/DNA-BC.

**Table 2:** Nucleotide sequence identity (%) among DNA sequences of cucurbit-infecting begomovirus in Malaysia.

Virus Cluster ^1^	Virus Species ^2^	Cluster 1a	Cluster 2a	Cluster 3a	Cluster 4a	Cluster 5a	Cluster 6a
Cluster 1a	ToLCNDV	92.5–98.9					
Cluster 2a	ToLCNDV	90.2–95.8	93.9–99.5				
Cluster 3a	ToLCNDV	88.4–92.9	88.1–91.1	99.0–100			
Cluster 4a	ToLCNDV	89.4–94.5	88.9–92.9	88.0–90.1	94.5		
Cluster 5a	SLCCNV					92.9–96.1	
Cluster 6a	SLCCNV					89.6–94.4	91.6–100.0
Other ToLCNDV DNA-A		78.3–86.3	78.3–85.6	78.9–86.7	80.8–88.0	73.0–79.9	73.4–79.7
		**Cluster 1b**	**Cluster 2b**	**Cluster 3b**	**Cluster 4b**	**Cluster 5b**	**Cluster 6b**
Cluster 1b	ToLCNDV	92.0–99.6					
Cluster 2b	ToLCNDV	84.9–91.4	92.5–100				
Cluster 3b	SLCCNV			85.8–88.5			
Cluster 4b	SLCCNV			73.9–77.5	84.3		
Cluster 5b	SLCCNV			75.6–85.1	77.5–80.3	97.3	
Cluster 6b	SLCCNV			70.9–80.8	75.9–82.3	80.8–82.5	83.9–99.7
Other ToLCNDV DNA-B		78.2–79.4	74.6–77.4	54.8–62.2	55.3–55.4	54.8–55.4	54.8–55.0

^1^ The virus cluster was based on the phylogenetic tree in Figure 1. The DNA sequences of ToLCNDV and SLCCNV retrieved from GenBank, listed in the Figure 1 and Table S2 were included in the analysis; ^2^ ToLCDNV: *Tomato leaf curl New Delhi virus*; SLCCNV: *Squash leaf curl China virus*.

**Table 3:** Pathogenicity of infectious cucurbit-infecting begomoviral DNAs from Malaysia.

Crops	SLCCNV-A [MY-Sq3-5-16]		SLCCNV-A [MY-Sq115-17]		SLCCNV-A [MY-Sq157-17]		ToLCNDV-A [MY-OM1-16]		ToLCNDV-B [MY-Wax12-16]		ToLCNDV-D [MY-BG85-17]		ToLCNDV-A [MY-BG120-17]		ToLCNDV-B[MY-Wax12-16] DNA-A + SLCCNV-A[MY-Sq3-5-16] DNA-B	
	Symptom ^1^	PCR ^2^	Symptom	PCR	Symptom	PCR	Symptom	PCR	Symptom	PCR	Symptom	PCR	Symptom	PCR	Symptom	PCR
Bottle gourd	5/5	5/5	5/5	5/5	5/5	5/5	5/5	5/5	5/5	5/5	5/5	5/5	5/5	5/5	5/5	5/5
Cucumber	0/5	3/5	0/5	2/5	0/5	0/5	5/5	5/5	5/5	5/5	5/5	5/5	3/4	3/4	3/5	3/5
Oriental melon	5/5	5/5	4/5	4/5	3/5	3/5	4/5	4/5	4/5	4/5	2/2	2/2	3/5	3/5	0/5	0/5
Pumpkin	12/15	12/15	2/5	2/5	2/5	2/5	8/15	8/15	8/15	8/15	1/5	1/5	4/5	4/5	13/18	13/18
Squash	8/8	8/8	5/5	5/5	5/5	5/5	0/8	0/8	0/8	0/8	0/5	0/5	0/5	0/5	0/13	3/13
Wax gourd	4/4	4/4	4/4	4/4	4/4	4/4	4/4	4/4	4/4	4/4	3/4	3/4	4/4	4/4	4/4	4/4
Tobacco	0/7	7/7	0/5	5/5	0/5	5/5	7/7	7/7	7/7	7/7	5/5	5/5	5/5	5/5	7/7	7/7
Tomato	0/5	0/5	0/5	0/5	0/5	0/5	0/5	1/5	0/5	1/5	0/5	0/5	5/5	5/5	9/10	9/10

^1^ Symptomatic plants/tested plants were observed for four weeks after agroinoculation; ^2^ PCR positive plants/tested plants were conducted by PCR using the primers PAL1v1978RYNN and PAR1c715H at four weeks after agroinoculation.

## Data Availability

The data presented in this study are openly available in National Center for Biotechnology Information, U. S. National Library of Medicine, USA.

## Supplementary Materials

The following are available online at https://www.mdpi.com/article/10.3390/plants10112396/s1, Figure S1: 1.2% agarose gel electrophoresis of specific detection of cucurbit-infecting begomoviruses in Malaysia, Table S1: Sequences of primers used in this study, Table S2: Begomovirus DNA sequences used in this study, Table S3: Characteristics of full-length DNA sequences of cucurbit-infecting begomoviruses collected in Malaysia in 2016–2017.
