# Supplementary material for: Genetic Diversity, Pathogenicity and Pseudorecombination of Cucurbit-Infecting Begomoviruses in Malaysia
Source: Plants (Basel). 2021 Nov 6;10(11):2396. doi: 10.3390/plants10112396 (PMC8624487; doi:10.3390/plants10112396)
Supplement: Supplementary file 1 [file plants-10-02396-s001.zip › MS-plants (3) Figure S1.pdf]

plants

MDPI

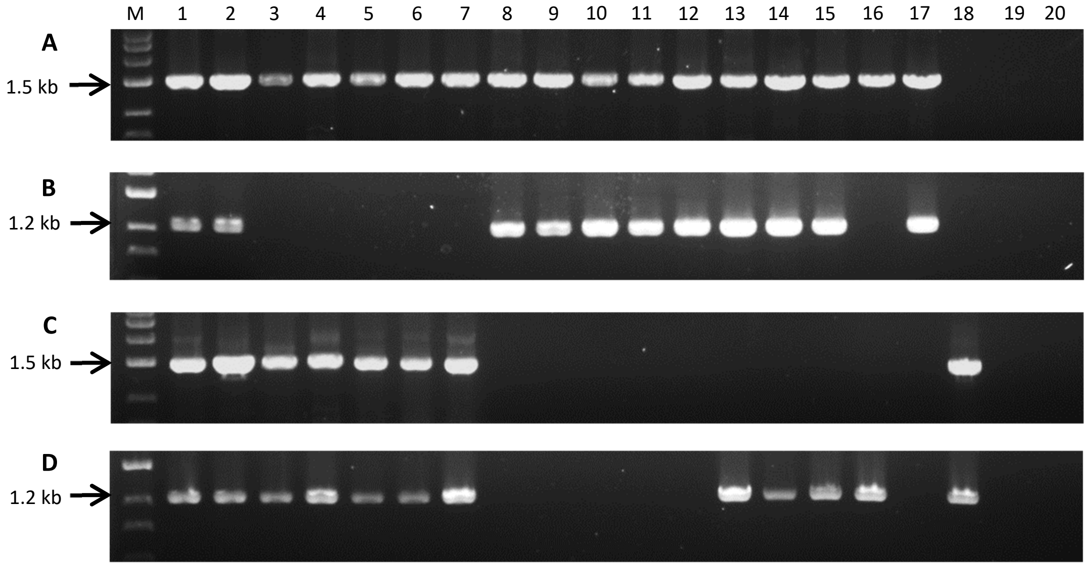

Figure S1. 1.2% agarose gel electrophoresis of specific detection of cucurbit-infecting begomoviruses in Malaysia. The virus specific detection was conducted by PCR using the specific primer pair-ToLCNDV-SPAF/PAR1c715H for the *Tomato leaf curl New Delhi virus* (ToLCNDV) DNA-A (A), the specific primer pair-ToLCNDV-SPBF/DNA-BC for the ToLCNDV DNA-B (B), the specific primer pair-SLCCNV-SPAF/PAR1c715H for the *Squash leaf curl China virus* (SLCCNV) DNA-A (C), and the specific primer pair-SLCCNV-SPBF/DNA-BC for the SLCCNV DNA-B (D). Lane M is the DNA molecular 1kb size marker. Lanes 1 to 16 are viral DNAs extracted from symptomatic cucurbit leaf samples Sq112, Sq158, Sq3, BoG5, Sq115, Sq157, Sq107, OM1, Wax12, Cu146, RG14, Cu63, BG85, BG100, BG166 and BG120. Lane 17 is viral DNA extracted from a ToLCNDV-infected pumpkin plant. Lane 18 is viral DNA extracted from a SLCCNV-infected pumpkin plant. Lane 19 is a healthy squash. Lane 20 is a buffer control.
