# Supplementary material for: Genetic Diversity, Pathogenicity and Pseudorecombination of Cucurbit-Infecting Begomoviruses in Malaysia
Source: Plants (Basel). 2021 Nov 6;10(11):2396. doi: 10.3390/plants10112396 (PMC8624487; doi:10.3390/plants10112396)
Supplement: Supplementary file 1 [file plants-10-02396-s001.zip › MS-plants (3) Table S1.pdf]

plants

MDPI

Table S1. Sequences of primers used in this study.

Primers	Sequence (5′ to 3′)	Purpose	References
PAL1v1978B	GCATCTGCAGGCCCACATBGTYTTHCCNGT	General DNA-A detection of begomoviruses	Tsai *et al*., 2011
PAR1c715H	GATTTCTGCAGTTDATRTTHTCRTCCATCCA	General DNA-A detection of begomoviruses	Tsai *et al*., 2011
PAL1v1978RYNN	GCATCTGCAGGCCCACRYNGTYTTNCCNGT	General DNA-A detection of begomoviruses	This study
DNA-BV	ACAATAYRCRCGTAAGSAAATMTGTGA	General DNA-B detection of begomoviruses	This study
DNA-BC	TCTSMAAGGATAARAATCCTTGGA	General DNA-B detection of begomoviruses	This study
SLCCNV-SPAF	GGWCCYAAHGAMCGMGCCCACGC	SLCCNV DNA-A specific detection	This study
ToLCNDV-SPAF	GKKCCTAWRSAMCGAGCCCACAT	ToLCNDV DNA-A specific detection	This study
SLCCNV-SPBF	GTYCGTTCATATATCAARCTMACA	SLCCNV DNA-B specific detection	This study
ToLCNDV-SPBF	GTTYGKTMBTATRTCAARYTRAAG	ToLCNDV DNA-B specific detection	This study
16MY3-3IFAV	ACCAGATCTAGGTGGCCGCACAAG	Full-length DNA-A amplification of 16MY3, 16MY5, 17MY85	This study
16MY3-3IFAC	ACCAGATCTAAGCCCGAGAGTGTAC	Full-length DNA-A amplification of 16MY3, 16MY5, 17MY85	This study
16MY09-FAV	ACACTGCAGAACATAATCTTTTGG	Full-length DNA-A amplification of 16MY1, 16MY9, 16MY10, 16MY12, 17MY120	This study
16MY09-FAC	CGCCTGCAGTTTCATAATTTGAA	Full-length DNA-A amplification of 16MY9, 16MY12	This study
16MY10-FAC	CGCCTGCAGTTTCATAATTTAAT	Full-length DNA-A amplification of 16MY10, 17MY120	This study
16MY1-3-FAC	CGCCTGCAGTTTCATAATCTAAG	Full-length DNA-A amplification of 16MY1	This study
FAV1	ACAGGATCCACAAACATGTGGGATC	Full-length DNA-A amplification of ToLCNDV isolates	This study
FAC1	GCCGGATCCAAACTTGGTGAGCAAGTCT	Full-length DNA-A amplification of ToLCNDV isolates	This study
17MY15-2FAV	ACAGGGTCCACAAACATGTGGGATC	Full-length DNA-A amplification of 17MY15	This study
17MY15-2FAC	GCCGGACCCAAACTTGGTGAGCAAGTCT	Full-length DNA-A amplification of 17MY15	This study
17MY115SLCCNV-FAV	ACTGGATCCACTTATGCACGAGTTTC	Full-length DNA-A amplification of 17MY115	This study
17MY115SLCCNV-FAC	CTCGGATCCCACATGTTGTGGTTCA	Full-length DNA-A amplification of 17MY115, 17MY157	This study
17MY157SLCCNV-FAV	ACTGGATCCACTTATGCACGACTTTC	Full-length DNA-A amplification of 17MY157	This study
17MY85ToLCNDV-FAV	TGCGTCGACGAGTTCTGGAGGAACTCT	Full-length DNA-A amplification of 17MY85	This study
17MY85ToLCNDV-FAC	TGAGTCGACTGGGCCAATGTAAATGT	Full-length DNA-A amplification of 17MY85	This study
17MY100-3FAC	GCCGGATCCGAACTTGTTGAGCAAGTCT	Full-length DNA-A amplification of 17MY102	This study
17MY132-1FAV	ACATGATCCACAAACATGTGGGATC	Full-length DNA-A amplification of 17MY132	This study
17MY132-1FAC	GCCGGATCAAAACTTGGTGAGCAAGTCT	Full-length DNA-A amplification of 17MY132	This study
17MY140-3FAC	GCCGGATCCGAACTTGGTGAGCAAGTCT	Full-length DNA-A amplification of 17MY63, 17MY115, 17MY120, 17MY137, 17MY140	This study
17MY146A-FAV	TAGCTGCAGATCAACTCGCCTCCTG	Full-length DNA-A amplification of 17MY146, 17MY157	This study
17MY146A-FAC	TAGCTGCAGTGTGGGTGCACG	Full-length DNA-A amplification of 17MY146, 17MY157	This study
16MY1-1FBV	GCACCATGGCAAATCACTCTCTCCAAC	Full-length DNA-B amplification of 16MY1, 16MY12	This study
16MY1-1FBC	GAACCATGGATACTTCGATTGCGCT	Full-length DNA-B amplification of 16MY1,16MY9, 16MY10, 16MY12	This study
16MY8-4FBV	GCACCATGGCAAATCACTTTCTCCAAC	Full-length DNA-B amplification of 16MY9	This study
16MY10-4FBV	GCACCATGGCAAATCACTCTCCCCAAC	Full-length DNA-B amplification of 16MY10	This study
16MY3PstI-FBV	CGCCTGCAGGATAATTTCGTTTCTAACGA	Full-length DNA-B amplification of 16MY3	This study
16MY3PstI-FBC	GATCTGCAGCTCTTCTAACACAGT	Full-length DNA-B amplification of 16MY3	This study
16MY5PstI-FBV	CGCCTGCAGGATAATTTCGTTTTTAACGA	Full-length DNA-B amplification of 16MY5	This study
16MY5PstI-FBC	GATCTGCAGCGCTTCTAACACAGT	Full-length DNA-B amplification of 16MY5	This study
16MY3-FBV	GATGAATTCGACGTCAGTCGAATG	Full-length DNA-B amplification of 16MY3	This study
17MY1B-FBC	CAGGAATTCCGACCACCAAAGATAG	Full-length DNA-B amplification of 16MY1	This study
17MY3B-FBC	CAGGAATTCCGACCACCGAAGATAG	Full-length DNA-B amplification of 16MY3	This study
17MY85B-FBC	CAGGAATTCCGAGCACCAAGAATAG	Full-length DNA-B amplification of 16MY85	This study
17MY85SLCCNV-FBV	CAGCCATGGATTGATGCGTTATCG	Full-length DNA-B amplification of 17MY85, 17MY115	This study
17MY85SLCCNV-FBC	CGACCATGGTATTTCCGAGATCTC	Full-length DNA-B amplification of 17MY85, 17MY115, 17MY157	This study
17MY107B-FBC	CAGGAATTCCGAGCACCACGAATAG	Full-length DNA-B amplification of 17MY107,	This study
17MY120B-FBC	CAGGAATTCCGAGCACCCAGAATAG	Full-length DNA-B amplification of 17MY120	This study
17MY157-FBV	GCCAGATCTCGGAAATACCATGG	Full-length DNA-B amplification of 17MY157	This study
17MY157-FBC	TGCAGATCTCCAAAACTTCAATTTC	Full-length DNA-B amplification of 17MY157	This study
16MY1-1FBV	GCACCATGGCAAATCACTCTCTCCAAC	Full-length DNA-B amplification of 17MY85, 17MY30, 17MY63, 17MY112, 17MY140, 17MY146	This study
16MY1-1FBC	GAACCATGGATACTTCGATTGCGCT	Full-length DNA-B amplification of 17MY85, 17MY30, 17MY63, 17MY112, 17MY140, 17MY146	This study

B=C, G, T; D=A, G, T; H=A, C, T; K=G, T; M=A, C; N=A, T, C, G; R=A, G; S=C, G; V=A, C, G; W=A, T; Y=C, T
