# Supplementary material for: Genetic Diversity, Pathogenicity and Pseudorecombination of Cucurbit-Infecting Begomoviruses in Malaysia
Source: Plants (Basel). 2021 Nov 6;10(11):2396. doi: 10.3390/plants10112396 (PMC8624487; doi:10.3390/plants10112396)
Supplement: Supplementary file 1 [file plants-10-02396-s001.zip › MS-plants (3) Table S2.pdf]

plants

MDPI

Table S2. The sequences of begomoviral DNAs used in this study.

Viruses	Abbreviation	GenBank accession	
		DNA-A	DNA-B
Squash leaf curl China virus MC1 cucumber isolate from Malaysia	SLCCNV-[MY-MC1]	EF197940	
Squash leaf curl China virus squash isolate from China	SLCCNV-[CN-Sq-17]	MG525551	MG525552
Squash leaf curl China virus P54 chayote isolate from Philippines	SLCCNV-[PH-P54]	EU487031	
Squash leaf curl China virus B isolate of cucurbits in Vietnam	SLCCNV-[VN-B]	AF509743	AF509742
Squash leaf curl China virus wax gourd isolate from Thailand	SLCCNV-[TH-Wax]	EU543562	
Squash leaf curl China virus Hanoi isolate of squash from Vietnam	SLCCNV-[VN-Sq-12]	KC857509	KC857510
Squash leaf curl China virus squash isolate from Indonesia	SLCCNV-[ID-BASq-17]	LC511776	LC511781
Tomato leaf curl New Delhi virus luffa isolate from Indonesia	ToLCNDV-[IN-JV-Luf-17]	LC431619	LC431620
Tomato leaf curl New Delhi virus cucumber isolates from Indonesia	ToLCNDV-[ID-BACu-20]	LC511775	LC511780
Tomato leaf curl New Delhi virus bitter gourd isolate from Pakistan	ToLCDNV-[PK-Mn-05]	AM747291	
Tomato leaf curl New Delhi virus luffa isolate from Thailand	ToLCNDV-[TH-Luffa-98]	AF102276	
Tomato leaf curl New Delhi virus bitter gourd isolate from India	ToLCDNV-[IN-BTG-1]	KY780207	KY780208
Tomato leaf curl New Delhi virus 4 isolate TC306 from India	ToLCDNV4-[IN-TC306-11]	KF551592	
Squash leaf curl Philippines virus isolates AFPK5slv from Taiwan	SLCuPV-[TW-PK5]	EF199774	
Squash leaf curl Philippines virus pumpkin isolate from Taiwan	SLCuPV-[TW-PA1]	DQ866135	
Squash leaf curl Philippines virus wax gourd isolate from Taiwan	SLCuPV-[TW-Wg1]	EU310406	
Squash leaf curl Philippines virus Yunling isolate in Taiwan	SLCuPV-[TW-YL]		EU479711
Squash leaf curl Philippines virus pumpkin isolate from Philippines	SLCuPV-[PH-Sq]	AB085793	AB085794
Pumpkin yellow mosaic Malaysia virus pumpkin isolate from Malaysia	PuYMV-[MY-MP1-01]	EF197941	
Squash leaf curl Yunnan virus isolate 23 from China	SLCuYV-[CN-Y23]	AJ420319	
Squash leaf curl virus	SLCuV-[US-Sq]	M38183	M38182
