# Supplementary material for: Genetic Diversity, Pathogenicity and Pseudorecombination of Cucurbit-Infecting Begomoviruses in Malaysia
Source: Plants (Basel). 2021 Nov 6;10(11):2396. doi: 10.3390/plants10112396 (PMC8624487; doi:10.3390/plants10112396)
Supplement: Supplementary file 1 [file plants-10-02396-s001.zip › MS-plants (3) Table S3.pdf]

plants

Table S3. Characteristics of full-length DNA sequences of cucurbit-infecting begomoviruses collected from Malaysia in 2016-2017.

Province	Location	Field No.	No.	DNA-A ORF regions, nt									DNA-B ORF regions, nt				
				Length, nt	IR	AV2 (Pre)	AV1 (CP)	AC1	AC2	AC3	AC4	Accessions	Length, nt	IR	NSP (BV1)	MP (BC1)	Accessions
Sarawak	Sematan	1	ToLCNDV-A[MY-OM1-16]	2739	2585-119	120-458	280-1050	2584-1499	1596-1177	1457-1047	2427-2251	MT912475	2690	2150-441	442-1248	2149-1304	MT912476
		2	SLCCNV-A[MY-Sq3-5-16]	2738	2585-119	120-458	280-1050	2584-1499	1596-1192	1457-1047	2427-2251	MW248679	2661	2154-446	447-1253	2153-1308	MW248681
		3	SLCCNV-A[MY-BoG5-16]	2738	2585-119	120-458	280-1050	2584-1499	1596-1192	1457-1047	2427-2251	MW248682	2663	2156-448	449-1255	2155-1310	MW248683
	Kuching	4	ToLCNDV-B[MY-RG9-16]	2737	2584-118	119-457	279-1049	2583-1498	1595-1191	1456-1046	2426-2250	MW248639	2680	2141-432	433-1239	2140-1295	MW248640
		5	ToLCNDV-B[MY-Cu10-16]	2738	2585-119	120-458	280-1050	2584-1499	1596-1192	1457-1047	2427-2251	MW248641	2689	2150-441	442-1248	2149-1304	MW248642
		6	ToLCNDV-B[MY-Wax12-16]	2738	2585-119	120-458	280-1050	2584-1499	1596-1192	1457-1047	2427-2251	MW248643	2688	2150-441	442-1248	2149-1304	MW248644
		7	ToLCNDV-B[MY-Cu1-17]	2737	2584-119	120-458	280-1050	2583-1615	1596-1192	1457-1047	2426-2250	MW248645	2689	2150-441	442-1248	2149-1304	MW248646
		8	ToLCNDV-B[MY-RG11-17]	2738	2585-119	120-458	280-1050	2584-1499	1596-1192	1457-1047	2427-2251	MW248647	2688	2149-440	443-1249	2149-1304	MW248648
			ToLCNDV-B[MY-RG15-17]	2739	2585-119	120-458	280-1050	2584-1499	1596-1192	1457-1047	2427-2251	MW248649	2689	2150-441	442-1248	2149-1304	MW248650
		10	ToLCNDV-B[MY-Cu63-17]	2739	2585-119	120-458	280-1050	2584-1499	1596-1192	1457-1047	2427-2251	MW248651	2691	2269-443	444-1250	2268-1306	MW248652
Johor	Kulai	1	ToLCNDV-D[MY-BG85-17] [1]	2739	2585-119	120-458	280-1050	2584-1499	1596-1177	1457-1047	2427-2251	MW248653	2691	2084-440	441-1247	2083-1304	MW248654
			SLCCNV-D[MY-BG85-17] [1]										2686	2166-459	460-1266	2165-1320	MW248684
		3	SLCCNV-A[MY-Sq107-17]	2737	2585-119	120-458	280-1050	2584-1499	1596-1192	1457-1047	2427-2251	MW248685	2687	2177-584	585-1277	2176-1331	MW248686
			ToLCNDV-A[MY-Sq112-17]	2738	2585-119	120-458	280-1050	2584-1499	1596-1177	1457-1047	2427-2251	MW248655	2689	2149-439	440-1246	2148-1303	MW248656
			SLCCNV-A[MY-Sq115-17]	2737	2585-119	120-458	280-1050	2584-1499	1596-1192	1457-1047	2427-2251	MW248687	2688	2177-470	471-1277	2176-1331	MW248688
			ToLCNDV-A[MY-BG120-17]	2738	2585-119	120-458	280-1050	2584-1499	1596-1177	1457-1047	2427-2251	MW248677	2364	2059-466	467-1273	2058-1327	MW248678
		4	ToLCNDV-A[MY-Wax132-17]	2739	2586-120	121-459	281-1051	2585-1500	1597-1178	1458-1048	2428-2252	MW248657	2690	2150-440	441-1247	2149-1304	MW248658
		5	ToLCNDV-A[MY-Cu135-17]	2737	2584-119	120-458	280-1050	2583-1498	1625-1176	1456-1064	2426-2250	MW248659	2691	2150-440	441-1247	2149-1580	MW248660
			ToLCNDV-A[MY-Wax137-17]	2737	2585-119	120-458	280-1050	2584-1499	1551-1177	1457-1047	2427-2251	MW248661	2690	2150-440	441-1247	2149-1304	MW248662
		6	ToLCNDV-A[MY-Cu140-17]	2739	2585-119	120-458	280-1050	2584-1499	1596-1177	1457-1047	2427-2251	MW248663	2691	2269-443	444-1250	2268-1306	MW248664
			ToLCNDV-A[MY-Cu143-17]	2738	2585-119	120-458	280-1050	2584-1499	1596-1177	1457-1047	2427-2251	MW248665	2685	2150-440	441-1247	2149-1304	MW248666
			ToLCNDV-C[MY-Cu146-17]	2730	2585-119	120-458	280-1011	2584-1499	1596-1177	1457-1047	2427-2251	MW248667	2690	2150-392	393-1247	2149-1304	MW248668
		7	ToLCNDV-A[MY-Cu149-17]	2736	2583-120	121-459	281-1051	2582-1500	1594-1178	1458-1048	2425-2249	MW248669	2691	2150-440	441-1247	2149-1304	MW248670
			ToLCNDV-C[MY-Cu150-17]	2730	2585-119	120-458	280-1050	2584-1499	1596-1177	1457-1047	2427-2251	MW248671	2691	2150-440	441-1247	2149-1304	MW248672
			ToLCNDV-A[MY-Cu152-17]	2738	2585-119	120-458	280-1050	2584-1499	1596-1177	1457-1047	2427-2284	MW248673	2690	2150-440	441-1247	2149-1304	MW248674
		8	SLCCNV-A[MY-Sq157-17]	2737	2585-119	120-458	280-1050	2584-1499	1596-1192	1457-1047	2427-2251	MW248689	2723	2180-473	474-1280	2179-1334	MW248690
		9	ToLCNDV-A[MY-BG167-17]	2738	2585-119	120-458	280-1050	2584-1499	1596-1192	1457-1047	2427-2251	MW248675	2690	2150-440	441-1247	2149-1304	MW248676

[1] Two begomovirus DNA-Bs were identified. ToLCNDV: *Tomato leaf curl New Delhi virus*; SLCCNV: *Squash leaf curl China virus*.
